# Time Does Matter: The Cellular Response to Resveratrol Varies Depending on the Exposure Duration

**DOI:** 10.3390/ijms26125542

**Published:** 2025-06-10

**Authors:** Michalina Gramatyka

**Affiliations:** Maria Skłodowska-Curie National Research Institute of Oncology, Gliwice Branch, Wybrzeże Armii Krajowej 15, 44-101 Gliwice, Poland; michalina.gramatyka@gliwice.nio.gov.pl

**Keywords:** resveratrol, cardioprotection, dietary compounds, energy metabolism

## Abstract

Resveratrol is a natural polyphenol found in grapes, berries, and red wine, commonly studied for its biological activity. In vitro research often uses high concentrations of resveratrol applied for short incubation times. However, resveratrol reaches relatively low concentrations in vivo when it is used as a dietary supplement. Therefore, the aim of this study was to investigate the cellular response of cardiomyocytes to low, physiologically relevant concentrations of resveratrol and, in particular, to compare these responses depending on the duration of exposure. Cardiomyocytes were treated with resveratrol for either 1 day, 1 week, or 1 month. Functional assays assessing metabolic activity, cell cycle distribution, and apoptosis intensity were performed, along with analysis of selected pathways at protein levels. The results showed that the cellular response differed markedly depending on the duration of resveratrol treatment. Observed changes indicated alterations in energy metabolism and effects consistent with anti-aging activity.

## 1. Introduction

Phenolic compounds are a large group of chemicals, usually of plant origin, that possess various properties. They serve as scents, pigments, feeding deterrents, antifungal and antimicrobial agents, poisons, signaling molecules, or structural components [1]. Due to the properties of polyphenols—primarily antioxidative—there is growing interest in using them as dietary ingredients that could improve health or prevent the development of many diseases (chemoprevention). The presence of polyphenols, including flavonoids, in the diet is considered to have beneficial effects on the cardiovascular system, nervous system, immune response, gut microflora, and even the course of bacterial and viral infections, and has been extensively characterized in numerous papers [1,2,3,4,5].

Among all plant polyphenols, resveratrol (3,5,4′-trihydroxystilbene) raises great interest as a potential chemopreventive and chemoprotective dietary compound. Resveratrol is a polyphenol from stilbene class, also classified as phytoestrogen [6,7]. It naturally occurs in red grapes, nuts or berries [3,6,8], and functions as a phytoalexin—a substance synthesized by plants in response to pathogen infection [3,6]. Numerous papers relating to resveratrol suggest that it protects against oxidative stress, inflammation, cancer, neurological diseases, diabetes, obesity, and even against ageing [3,6,7,8,9,10,11,12,13,14,15,16]. It may also possess protective properties in the context of chemotherapy and radiotherapy [17,18,19,20,21,22]. Several studies also describe the beneficial effects of resveratrol on the cardiovascular system, pointing to its anti-atherosclerotic and anticoagulant properties, as well as its ability to reduce blood pressure, the size of heart infarction, and even the cardiotoxicity of anticancer treatment [3,11,23,24]. Long-term resveratrol supplementation may also improve cognitive function in older adults [25].

Many papers describe the protective properties of resveratrol, both in vitro and in vivo. However, in in vitro studies, the incubation of cells with resveratrol usually lasts for a relatively short time. Also, researchers frequently use high doses, unattainable in the in vivo systems [8,13,20,26]. Considering resveratrol as a dietary component, in vitro studies investigating the molecular mechanisms of resveratrol action should put more focus on long-term administration in small doses, an approach partially supported by the literature [16,27,28,29]. Despite this, there is a substantial lack of research that considers these important factors, and the majority of published studies may be irrelevant for explaining the mechanisms of resveratrol action in the human body. Accordingly, the purpose of this study was to evaluate whether and how different incubation times with the same, low dose of resveratrol would influence the cells cultured in vitro. Human cardiomyocytes were selected as an experimental model because several studies indicated potential cardioprotective activity of this compound.

## 2. Results

**Figure 1:**
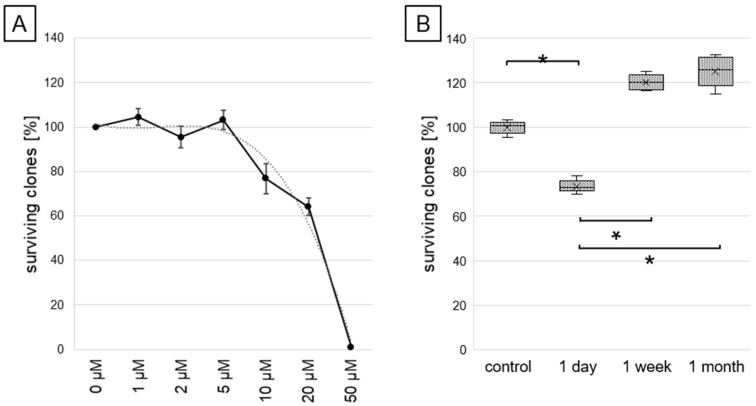
Assessment of the inhibitory concentration of resveratrol for human cardiomyocytes (**A**) and the influence of different incubation times at resveratrol concentration 5 µM on clonogenic survival of cardiomyocytes (**B**). Numbers of surviving clones are presented in relation to untreated controls (100%); statistically significant differences are marked with asterisks (*p* < 0.05).

In this study, it was assessed how different exposure times to resveratrol influence cardiomyocytes’ viability and activity of selected molecular pathways. In pilot experiments, several doses of resveratrol (up to 50 µM) were tested to investigate their effect on cardiac cell survival. The effect of DMSO (used as a resveratrol solvent) on the clonogenic potential of cardiomyocytes was also examined. The half maximal inhibitory concentration (IC_50_) of resveratrol for cardiomyocytes was estimated as 25 µM (Figure 1A). DMSO had no adverse effect on clonogenic potential within the tested dose range. Based on these results and the available literature, a 5 µM concentration of resveratrol was selected for further studies, as this dose is achievable in in vitro systems (considering the fast degradation rate of resveratrol in the body) [30].

### 2.1. Cell Survival and Viability

The effect of resveratrol on the survival and metabolic activity of cardiomyocytes was evaluated. A standard colony-forming assay demonstrated that the number of colonies formed was decreased by incubation with resveratrol for 1 day, while this number was increased by longer exposure (for 1 week and 1 month), as shown in Figure 1B (differences were statistically significant).

**Figure 2:**
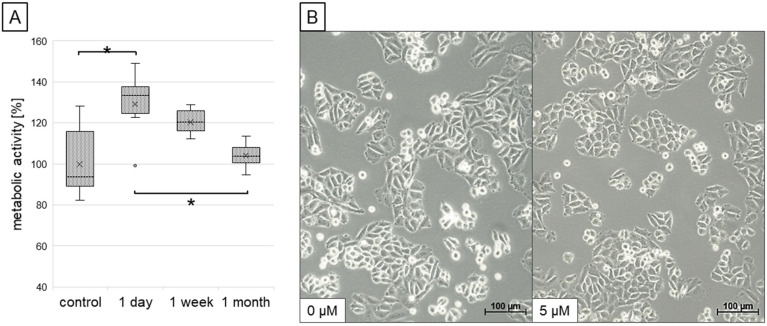
The influence of different incubation times with resveratrol on the mitochondrial metabolic activity of cardiomyocytes was measured using the XTT test (**A**). The relative activity is presented in relation to untreated controls (100%); statistically significant differences are marked with asterisks (*p* < 0.05). Morphology of control (0 µM) and resveratrol-treated (5 µM) cardiomyocytes observed under light microscopy (**B**).

The mitochondrial metabolic activity of cardiomyocytes was assessed by a simple colorimetric assay based on the conversion of tetrazolium salt to formazan (XTT assay), and results are shown in Figure 2A. It was observed that short exposure of cardiomyocytes to resveratrol (1 day) markedly increased the metabolic activity of mitochondria. In contrast, cells adapted to this compound after prolonged exposure (1 week and 1 month), and metabolic activity measured by this assay was similar to control cells. Notably, the treatment with resveratrol at low concentration used in the study (5 µM) did not affect the morphology of cardiomyocytes (Figure 2B).

**Figure 3:**
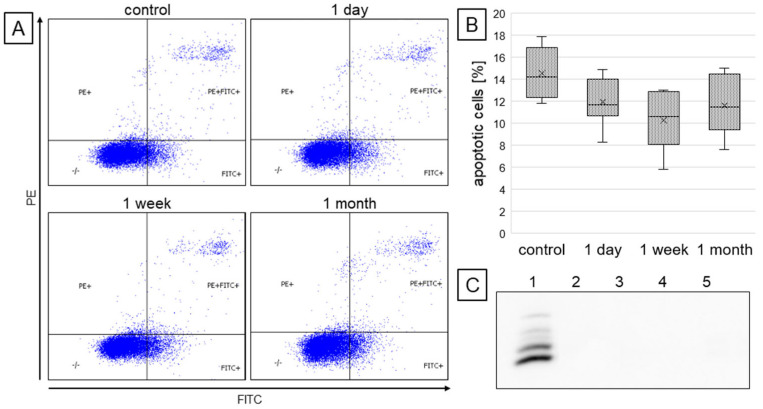
The influence of different incubation times with resveratrol on apoptosis of cardiomyocytes. Cells were stained with PI and anti-AnnexinV antibody, and analyzed by flow cytometry to estimate the number of apoptotic cells (PE and FITC-positive) (**A**,**B**). Activated caspase 3 was analyzed by Western blots using an antibody specific for the cleaved/activated form (**C**); lanes marked: 1—positive control (heat-induced apoptosis), 2—no resveratrol (control), 3—1 day, 4—1 week, 5—1 month of resveratrol treatment.

The annexin V-FITC and propidium iodide staining allowed for flow cytometric evaluation of the number of apoptotic cells in each study group. It was observed (Figure 3A,B) that treatment with resveratrol, independently of the incubation time, slightly reduced the number of apoptotic cells (i.e., Annexin V-positive cells), yet differences were not statistically significant. Moreover, Western blot analysis did not detect the active caspase 3 form in none of the groups tested (Figure 3C).

### 2.2. Changes in the Cell Cycle Phases Distribution

**Figure 4:**
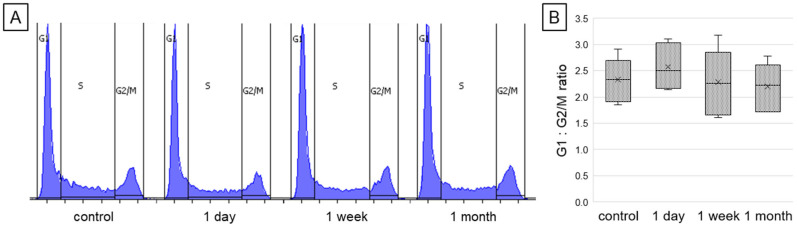
The influence of different incubation times with resveratrol on cell cycle phase distribution of cardiomyocytes. Numbers of cells in different phases were counted by flow cytometry and are presented as histograms showing the distribution of cell populations in different cycle phases (**A**), and the G1:G2/M ratio (**B**); the differences between tested groups were not statistically significant.

The potential effect of resveratrol on the distribution of cell cycle phases was assessed by flow cytometry-based analysis, and presented in Figure 4. This analysis revealed that exposure to resveratrol had no statistically significant effect on cell cycle arrest in either the G1 or G2/M phases, independently of exposure time. However, in the group treated for 1 day, a slight increase in the number of cells in the G1 phase was observed. The calculated G1:G2/M ratios in the studied groups were 2.3, 2.6, 2.3, and 2.2 for control, 1 day, 1 week, and 1 month of resveratrol treatment, respectively.

### 2.3. Changes in the Protein Expression

**Figure 5:**
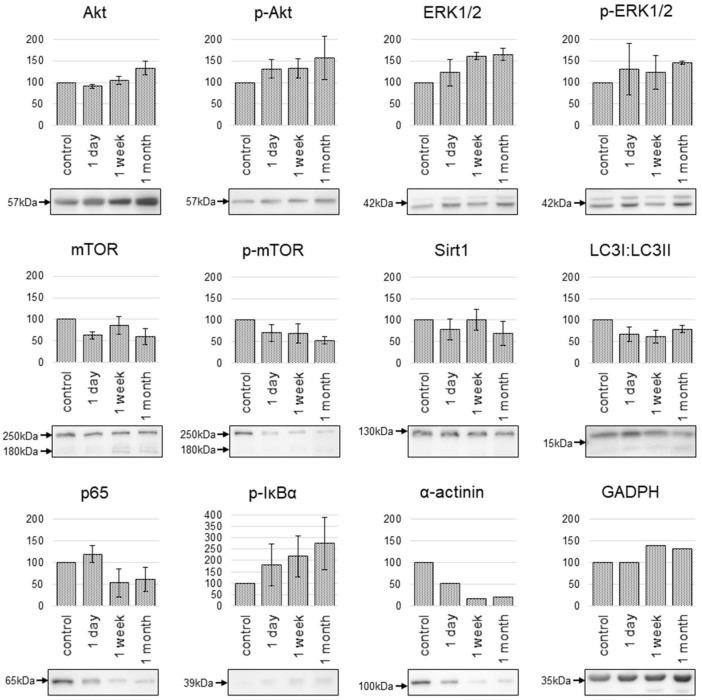
Changes in the levels of selected proteins in cardiomyocytes incubated for different times with resveratrol. Presented are representative Western blots and the relative quantification of analyzed proteins after normalization to β-actin (loading control) in relation to untreated controls (100%).

To address the activity of pathways reported to be relevant for response to resveratrol, the accumulation of proteins associated with these pathways was analyzed using Western-blots. Selected proteins included Sirt1, mTOR, Akt, ERK1/2, as well as proteins associated with the NFκB complex (IκBα, p65) and autophagy (LC3). The results are presented in Figure 5.

It was observed that resveratrol at longer incubation times (1 week and 1 month) increased the level of Akt and ERK proteins without affecting their phosphorylation levels (similar increase of total and phosphorylated forms). Moreover, resveratrol decreased mTOR protein levels (both total and phosphorylated forms), particularly after longer incubation times (1 week and 1 month). Interestingly, in samples of cells treated with resveratrol for a longer time, apart from the protein band of expected size (around 250 kDa), an increased level of a smaller protein form (about 180 kDa) was observed. It is noteworthy that, at the selected low concentration of resveratrol, the treatment did not affect Sirt1 protein levels, regardless of the incubation time.

Additionally, the level of IκBα phosphorylation increased proportionally to the incubation time with resveratrol, which indicated the possible activation of NFκB-related pathways. On the other hand, reduced levels of p65 protein observed after prolonged incubation time (1 week and 1 month) could reflect adaptation of cells to this compound. Furthermore, observed changes in the proportion of different forms of LC3 protein, and an increased level of LC3-II form in cells exposed to resveratrol (irrespective of the incubation time) indicated activation of autophagy. Surprisingly, prolonged exposure of cells to resveratrol (1 week and 1 month) had a noticeable effect on the levels of proteins often used as loading controls—GAPDH (increase) and α-actinin (decrease). These changes putatively reflected resveratrol-induced changes in cellular metabolism and stressed out the importance of a proper selection of “neutral” protein as a loading control for Western blot analyses.

## 3. Discussion

Since resveratrol is a potential dietary supplement, it seems reasonable to study its effects at low concentrations, achievable in the body after oral administration, and after prolonged treatment. The concentration of orally administered resveratrol may vary between the organs and tissues, but in the blood, it does not exceed 5 µM, and often is even lower [27,31]. Achieving higher concentrations of resveratrol in vivo is both difficult, since it is rapidly metabolized [6,7,32], and unfavorable, as resveratrol acts in hormesis and is toxic at high doses [14,33,34]. Prolonged exposure of cells to resveratrol more closely reflects its effects in the context of its use as a dietary compound, and enables better evaluation of its effects on cell and tissue structures and function compared to short, acute exposure [16,27], which is frequently underestimated in published studies. The results of this study clearly show that the response of human cardiomyocytes to low physiologically-relevant doses of resveratrol varies with exposure time. In basic tests for clonogenic capacity, viability, and even cell cycle phase distribution, the effect of resveratrol was noted after a short administration. However, these were not observed after prolonged treatment (one week and one month), which indicated the presence of adaptive mechanisms. In contrast, for most of the analyzed proteins relevant to known molecular mechanisms activated by resveratrol, stronger effects of resveratrol were observed after prolonged exposure.

Treatment of cardiomyocytes with resveratrol led to a transient (observed only after 1 day of incubation) increase in their mitochondrial metabolic activity, which was previously described [13,35,36,37]. The changes in the metabolic activity of cardiomyocytes, as well as the observed increase in Akt and phospho-Akt protein levels and the decrease in mTOR and phospho-mTOR expression, may be related to the activation of the AMPK signaling pathway by resveratrol. This is the known mechanism of action of this compound, responsible for preserving energy homeostasis [38,39]. AMPK activation results in both increased glucose uptake and metabolism, and increased beta-oxidation (among other things through Akt activation), as well as activation of autophagy through inhibition of mTOR activity [40,41,42]. The inhibition of mTOR expression and phosphorylation observed here is a frequently reported effect of resveratrol [31,38,43]. The mTOR protein exists in two independently functioning complexes responsible for controlling cell growth—mTORC1 and mTORC2. Activation of the AMPK pathway inhibits the activity of the mTORC1 complex, resulting in the activation of autophagy and suppression of transcription and translation [42]. Interestingly, activation of AMPK, independently of inhibition of the mTORC1 complex, leads to activation of the mTORC2 complex. The activated mTORC2 complex can additionally induce phosphorylation of the anti-apoptotic protein Akt [42], as observed in the results obtained in this study. The increase in mitochondrial metabolic activity was seen only after a short incubation time with resveratrol, while changes in protein expression also persisted after prolonged treatment. This suggests some ability of cells to adapt their metabolism to the altered conditions of protein expression.

Increased accumulation of LC3-II protein, as well as decreased level of activated mTOR protein, indicates an increase in autophagy in resveratrol-treated cells. This is also a frequently reported phenomenon in studies on this compound [11,43], as well as on other plant-derived polyphenols with similar molecular structures, such as curcumin [44]. Autophagy is a process essential for maintaining homeostasis in the cell: it is responsible for both removing damaged or unnecessary components of the cell, as well as maintaining energy production in nutrient deficiency [45]. Activation of autophagy in cardiomyocytes incubated with resveratrol may also be related to the anti-aging activity of resveratrol, that is suggested in the literature [31,35]. Inhibition of mTOR activity (through, for example, AMPK activation) is thought to be associated with increased lifespan in lower organisms, like yeast, and with delayed aging and protection against age-dependent diseases (such as cardiovascular disease, neurodegenerative diseases) in more complex organisms [38,46,47]. Inhibition of mTOR affects cellular pathways associated with processes linked to aging and leads to a reduction in mRNA translation (which favors protein homeostasis, e.g., during protein aggregation or oxidative stress), promotes autophagy (allowing aggregated proteins and damaged organelles to be removed from cells), creates improved conditions for the maintenance of stem cells, and reduces the number of aged cells [46]. Therefore, it is important to emphasize that these effects were observed primarily after prolonged exposure of cardiomyocytes to resveratrol. The noted increase in protein band at about 180 kDa in Western blots for mTOR and phospho-mTOR proteins may reflect nonspecific binding of both antibodies, but may also suggest truncation of the mTOR (or the appearance of additional splicing forms) due to the prolonged exposure to resveratrol.

In addition to activating autophagy, other mechanisms may also be responsible for the anti-aging effects of resveratrol. One such mechanism is the ability of resveratrol to inactivate the NF-κB complex and thereby the modulation of the NF-κB-related pathways [6,48,49]. NF-κB is a transcription factor responsible for cellular processes related to aging, such as cellular senescence, immune response, apoptosis, and regulation of metabolism. The literature indicates that increased NF-κB activity accelerates aging and is associated with some age-related diseases, such as Alzheimer’s disease and diabetes [47]. For this reason, reducing NF-κB activity could have an anti-aging effect. There are also studies showing that reduced levels of the p65 protein (which is a component of the NF-κB complex) in mice is associated with a slow-aging phenotype and a delay in the onset of age-related pathologies such as muscle atrophy and osteoporosis [47,50]. Moreover, a decrease in the amount of p65 protein was also observed in mice treated with rapamycin, which is an mTOR inhibitor [50]. This suggests that the ability of resveratrol to inhibit mTOR activity may be responsible for the decrease in p65 content in long-term resveratrol-treated cells in the present study. The observed decrease in p65 protein with a concomitant increase in IκBα phosphorylation may suggest increased degradation of IκBα (which would normally lead to nuclear translocation and activation of NF-κB complex), without an actual increase in transcriptional activation by the NF-κB complex. This topic would require further investigation.

Increased expression of the Sirt1 protein is, according to the literature, one of the mechanisms of action of resveratrol [18,38,39,51], and may be related to its anti-aging activity [7,11,47] and its ability to activate mitochondria, and may affect cell metabolism (among other ways, through the AMPK pathway) [39]. Sirt1 can also lead to the deacetylation of p65 and to a reduction of its levels in the cell, and decrease the activity of the NF-κB complex [47]. However, in this study, no significant changes in Sirt1 protein levels in cardiomyocytes were noted, regardless of the incubation time with resveratrol. It is possible that, in the low doses used in this study, resveratrol has no effect on Sirt1 levels, which is in contrast to other studies where markedly higher concentrations of resveratrol (usually exceeding 25 µM) were used [8,13,26,52,53]. Additionally, Sirt1 activation could be cell-type-specific (there are no published reports using cardiomyocytes as a model). There are also suggestions that Sirt1 activation is not specific to resveratrol, but to its metabolites [49]. However, the literature confirms that AMPK activation with mTOR inhibition in cells upon resveratrol exposure can occur independently of Sirt1 activation [38,51].

Different plant-derived polyphenols that showed cytoprotective effects (cardioprotection or radioprotection) are characterized by similar mechanisms of action. They can reduce oxidative stress (antioxidant activity), arrest or slow cell cycle progression, allowing for DNA damage repair, or interact with pathways regulating apoptosis and energy metabolism [54]. Resveratrol also affects cell cycle progression in a manner that is dependent on the dose, as well as the type of cells with which it interacts [6,8,17,55]. In this study, no statistically significant changes in the distribution of cell cycle phases were observed, regardless of the incubation time with resveratrol. However, a trend toward an increase in the population of cells in the G1 phase in the group treated with resveratrol for 1 day was visible. One of the cellular pathways responsible for regulating cell cycle progression during the G1 to S phase transition is the MAPK/ERK pathway [52,56]. Activation of this pathway—observed in this study as increased phosphorylation of the ERK1/2 protein—may also have an anti-apoptotic effect by upregulating the expression of pro-survival proteins such as Bcl-2, Bcl-xL, and MCL-1 [56]. The changes in apoptosis intensity observed in this study were not statistically significant, but suggested some reduction in the apoptotic cell population in the groups incubated with resveratrol.

An unexpected observation was the variation in the level of proteins often used as loading controls during Western blot analysis. The decrease in alpha-actinin following prolonged exposure to resveratrol may be related to resveratrol’s ability to influence cytoskeletal dynamics and cell migration potential [11,39]. GAPDH plays multiple roles in the cell, regulating both glucose metabolism and cell death, as well as microtubule interactions and membrane fusion [57]. It has also been suggested that GAPDH may play a role in telomere maintenance, which results in slowing cellular senescence [57]. In this context, the observed increase in the amount of GAPDH protein in long-term resveratrol-treated cells may have some correlation with the anti-aging activity of resveratrol, especially in the absence of concomitant increases in metabolic activity and cell death in groups treated with resveratrol for one week and one month.

Cardioprotective activity, including the ability to improve cardiac performance, is a well-documented property of resveratrol [3,8,11,14,16,33]. For this reason, cardiomyocytes were chosen for this study as a model for testing the long-term effect of resveratrol on the cardiovascular system. The obtained results could be further expanded by performing analyses on endothelial cells, which are also responsible for the proper functioning of blood vessels [3,49]. It would also be interesting to expand the study to evaluate the effects of different exposure times to resveratrol on the antioxidant capacity of cells. The literature indicates that such exposure increases the activation of antioxidant enzymes [11,49,58,59], but it is not known whether and how this effect depends on the incubation time.

## 4. Materials and Methods

Cell line and materials. Human cardiomyocytes were obtained from Celprogen (#1311001-09, Torrance, CA, USA) and cultured in flasks covered with fibronectin. Cells were cultured in DMEM/F12 medium supplemented with 10% FBS, 2 mM L-glutamine, and antibiotics, in a 37 °C humidified atmosphere containing 5% CO2. Trans-resveratrol (Sigma-Aldrich, Burlington, MA, USA) was dissolved in DMSO, and its stock solution (100 mM) was stored at −20 °C in the dark. Cells were incubated with 5µM resveratrol for 1 month, 1 week, or 1 day.

Colony forming assay. Cells were seeded into 6-well plates at a density of 1000 cells per well and then cultured for 10 days. The colonies were stained with a solution containing 0.5% crystal violet and 50% methanol, rinsed with water to remove excess dye, and then a number of colonies was counted.

Cell viability assay. XTT assay was used to determine cell viability and mitochondrial metabolic activity. Cells were seeded into 96-well plates. At an appropriate time point, the XTT reaction solution (Biological Industries, Kibbutz Beit-Haemek, Israel) was added, plates were incubated in an incubator (37 °C, humidified atmosphere, 5% CO_2_) for 3 h and absorbance was measured at wavelengths 470 nm and 630 nm.

Cell cycle analysis. To characterize a distribution of the cell cycle phases, cells were harvested by trypsinization and fixed with 70% ethanol. In collected cells, DNA was stained with PI/RNase Staining Buffer (BD Biosciences, Franklin Lakes, NJ, USA), and then analyzed by flow cytometry using a FACSCanto cytometer (Becton Dickinson, Franklin Lakes, NJ, USA).

Apoptosis analysis. To assess the intensity of cell death, the FITC Annexin V Apoptosis Detection Kit (BD Pharmingen, Franklin Lakes, NJ, USA) was used. In brief, cells were harvested by trypsinization, washed twice in PBS, and suspended in Binding Buffer. FITC Annexin V and propidium iodide were added to the cell suspensions and incubated in the dark for 15 min. Samples were analyzed by flow cytometry using FACSCanto cytometer (Becton Dickinson).

Western blot analysis. Changes in the levels of selected proteins were assessed by Western blot. In brief, cells were lysed with RIPA buffer, and protein concentration was determined using the Bradford method. Samples were separated on 6–15% SDS/PAGE gels and transferred onto nitrocellulose membrane. Membranes were incubated overnight at 4◦C with primary antibodies specific for mTOR (#2972S, Cell Signaling Technology, Danvers, MA, USA), phospho-mTOR (Ser2448) (#2971S, Cell Signaling Technology), ERK1/2 (#4696, Cell Signaling Technology), phospho-ERK1/2 (Thr202/Tyr204) (#9101, Cell Signaling Technology), Sirt1 (#13161-1-AP, Proteintech, Rosemont, IL, USA), Akt (#10176-2-AP, Proteintech), phospho-Akt (Ser473) (#66444-1-Ig, Proteintech) LC3 (#14600-1-AP, Proteintech), p65 (#66535-1-Ig, Proteintech), phospho-IκBα (Ser32/36) (#82349-1-RR, Proteintech), and cleaved-caspase 3 (Asp175) (#9664, Cell Signaling Technology). Next, membranes were incubated with the HRP-conjugated secondary antibody and developed with ECL detection reagents. The intensities of the bands on exposed films were quantified using Image Studio™ Lite software (version 5.2.5, LI-COR Biosciences, Lincoln, NE, USA).

Statistical analysis. All experiments were performed in triplicate at least. Significance of differences between compared groups was assessed by the Kruskal–Wallis test, followed by Dunn’s multiple comparison test. *p*-values below 0.05 were considered statistically significant.

## 5. Conclusions

This study demonstrates that the response of cardiomyocytes to low, physiologically achievable concentrations of resveratrol varies depending on the duration of exposure. The results suggest that molecular pathways activated by low concentrations of this compound were sustained and enhanced by prolonged exposure compared to the short-term treatment. Activated pathways were related to managing energy metabolism, and pro-survival pathways, and potentially exert anti-aging effects, putatively contributing to increasing the ability to overcome stress-induced adverse events (induced resilience).

## Data Availability

The raw data supporting the conclusions of this article will be made available by the authors on request.

## Abbreviations

The following abbreviations are used in this manuscript:

Akt	protein kinase B
AMPK	5′ adenosine monophosphate-activated protein kinase
Bcl-2	B-cell lymphoma 2 protein
Bcl-xL	B-cell lymphoma-extra large protein
DMSO	dimethyl sulfoxide
ERK1/2	extracellular signal-regulated kinase 1/2
GAPDH	glyceraldehyde-3-phosphate dehydrogenase
IC_50_	half-maximal inhibitory concentration
IκBα	inhibitor of nuclear factor kappa B alpha
LC3	microtubule-associated protein 1A/1B-light chain 3
MAPK	mitogen-activated protein kinase
MCL-1	myeloid cell leukemia-1 protein
mTOR	mammalian target of rapamycin
NF-κB	nuclear factor-kappa B
PI	propidium iodide
Sirt1	sirtuin 1
